# The critical role of apoptosis in mesenchymal stromal cell therapeutics and implications in homeostasis and normal tissue repair

**DOI:** 10.1038/s41423-023-01018-9

**Published:** 2023-04-25

**Authors:** Chiara Giacomini, Cecilia Granéli, Ryan Hicks, Francesco Dazzi

**Affiliations:** 1 School of Cardiovascular and Metabolic Medicine & Sciences, King’s College London, London, UK; 2 BioPharmaceuticals R&D Cell Therapy Department, Research and Early Development, Cardiovascular, Renal, and Metabolism (CVRM), BioPharmaceuticals R&D, AstraZeneca, Gothenburg, Sweden

**Keywords:** Mesenchymal stromal cells, Cell death, immunosuppression, Immunosuppression, Predictive markers

## Abstract

Mesenchymal stromal cells (MSCs) have been extensively tested for the treatment of numerous clinical conditions and have demonstrated good safety but mixed efficacy. Although this outcome can be attributed in part to the heterogeneity of cell preparations, the lack of mechanistic understanding and tools to establish cell pharmacokinetics and pharmacodynamics, as well as the poorly defined criteria for patient stratification, have hampered the design of informative clinical trials. We and others have demonstrated that MSCs can rapidly undergo apoptosis after their infusion. Apoptotic MSCs are phagocytosed by monocytes/macrophages that are then reprogrammed to become anti-inflammatory cells. MSC apoptosis occurs when the cells are injected into patients who harbor activated cytotoxic T or NK cells. Therefore, the activation state of cytotoxic T or NK cells can be used as a biomarker to predict clinical responses to MSC treatment. Building on a large body of preexisting data, an alternative view on the mechanism of MSCs is that an inflammation-dependent MSC secretome is largely responsible for their immunomodulatory activity. We will discuss how these different mechanisms can coexist and are instructed by two different types of MSC “licensing”: one that is cell-contact dependent and the second that is mediated by inflammatory cytokines. The varied and complex mechanisms by which MSCs can orchestrate inflammatory responses and how this function is specifically driven by inflammation support a physiological role for tissue stroma in tissue homeostasis, and it acts as a sensor of damage and initiator of tissue repair by reprogramming the inflammatory environment.

## Introduction

Mesenchymal stromal cell (MSC)-based therapies present promising immunosuppressive treatment options for a range of inflammatory diseases. However, their clinical success remains limited due to the incomplete understanding of the mechanism in vivo and the lack of criteria for patient selection.

The current view on MSC-mediated immunosuppression relies on extensive in vitro evidence demonstrating that MSCs can modulate the recipient immune system through the production of soluble factors. Furthermore, it is well documented that the environment to which MSCs are exposed shapes their therapeutic potential and that these cells require an inflammatory milieu to exert immunosuppressive effects. However, little is known about what happens to MSCs after they are administered to patients and how therapeutic effects can be achieved without MSC engraftment.

Recently, new insights have revealed that MSCs undergo extensive apoptosis within a few hours after infusion and are subsequently efferocytosed by local myeloid phagocytes, which are the ultimate players in the anti-inflammatory response. MSC apoptosis has therefore been recognized as a critical mechanism of immunosuppression in vivo, challenging the long-established hypothesis that viable cells are required for clinical efficacy. However, it remains unclear whether multiple mechanisms of action can take place simultaneously and whether viable and apoptotic cells contribute differently to the therapeutic benefit in different contexts.

Furthermore, the strong similarity of MSCs to tissue fibroblasts raises questions about whether stromal cell apoptosis could represent an innate mechanism to restore tissue homeostasis after injury. In this review, we summarize the current knowledge on the immunomodulatory and therapeutic properties of MSCs with a focus on MSC apoptosis as a key mechanism to elicit immunosuppression. We also propose the concept that stromal cell apoptosis may be important in orchestrating tissue repair responses by reprogramming the inflammatory environment.

## Mesenchymal stromal cells and immunosuppression

MSCs are a heterogeneous cell population of mesenchymal origin that can adhere to plastic and is characterized by the expression of a set of surface antigens. However, these criteria are not specific and cannot distinguish these cells from other stromal cell types, such as conventional tissue fibroblasts. Despite the ambiguity in their identification, MSCs have extensively been shown to exert potent immunosuppressive effects, and their use as advanced therapy medicinal products (ATMPs) is a matter of active investigation.

**Fig. 1:**
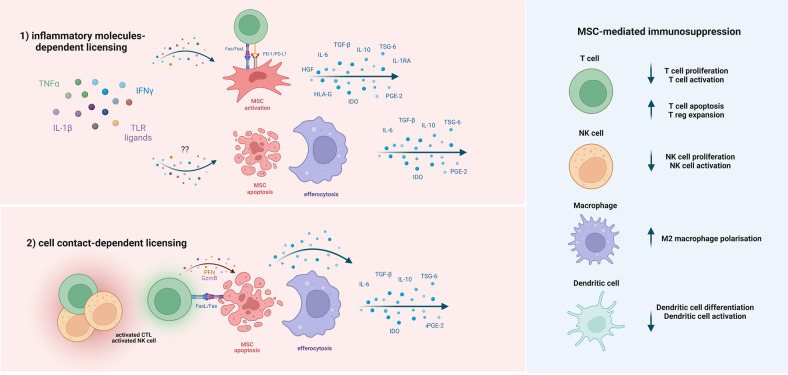
Mechanisms of MSC-mediated immunosuppression. Licensing of MSCs is essential to induce their immunosuppressive effects and can occur via stimulation with cytokines such as IFN-γ, TNF-α, IL-1β or TLR ligands present in the inflammatory microenvironment, or via direct cell–cell contact with activated cytotoxic T and NK cells. 1) MSCs licensed by soluble factors become potent immunomodulatory agents by producing an immunosuppressive secretome characterized by molecules such as IDO, PGE2, TGF-β, TSG-6, IL-10, IL-6, HGF, soluble HLA-G and IL-1RA and the activation of PD-1/PD-L1 and Fas/FasL signaling. In addition, the combination of soluble molecules present in the microenvironment can activate apoptotic pathways in MSCs and their subsequent efferocytosis by monocytes/macrophages. 2) MSCs licensed by cytotoxic cells undergo rapid caspase-dependent apoptosis and are efferocytosed by monocytes/macrophages, which become anti-inflammatory cells releasing similar immunosuppressive molecules. Apoptotic MSCs further contribute to immunosuppression by releasing caspase-dependent immunomodulatory factors. These licensing mechanisms result in efficient immunomodulation through the inhibition of T cells, NK cells, and DCs, M2 macrophage polarization and Treg expansion. Figure generated using BioRender.com

MSC-derived therapeutic activity is the result of the broad modulation of adaptive and innate immune cells. This intercellular communication is complex and multifactorial, and the signaling pathways involved are initiated indirectly through soluble factors and directly through cell–cell contact (Fig. 1).

MSCs, as well as all stromal cells, are characterized by intrinsic functional plasticity, which allows them to promptly adapt and respond to the surrounding microenvironment. When exposed to an inflammatory milieu, MSCs undergo functional reprogramming and become activated to exert their immunomodulatory functions, a process called “licensing” [1]. Several inflammatory molecules are responsible for this licensing step, including proinflammatory cytokines such as IFN-γ, TNF-α, and IL-1β, as well as Toll-like receptor (TLR) ligands.

By analyzing the effects of different TLR ligands, it has been proposed that MSCs can be polarized toward a mild proinflammatory phenotype (MSC1) in alternative to the more conventional anti-inflammatory profile (MSC2) adopting criteria similar to those used to functionally classify macrophages [2]. Earlier studies showed that TLR-3 activation induces MSCs to secrete anti-inflammatory molecules such as IL-10, indoleamine 2,3-dioxygenase (IDO), prostaglandin E2 (PGE2), and IL-1RA. In contrast, TLR-4 stimulation induces an MSC1-like secretome that includes IL-6, IL-8, and TGF-β [2–4]. However, the MSC1-like secretome consists of molecules that are only mildly proinflammatory, and later studies have failed to confirm these two profiles and showed different outcomes depending on factors such as the MSC source, TLR ligand concentration, the use of costimulatory molecules, and the in vitro assay used to measure the immunomodulatory activity. For example, the combination of TLR-3 and TLR-4 stimulation was reported to strongly inhibit or enhance MSC-mediated immunosuppressive effects on T-cell proliferation in different studies [5, 6]. This discrepancy could be ascribed to the differences in the culture conditions and in the length of MSC exposure to the stimuli. In another study, longer exposure to LPS increased the immunosuppressive capacity of MSCs in vitro and in vivo in a mouse model of experimental autoimmune encephalomyelitis [7]. Recent work suggested that TLR-3 activation in MSCs promoted regulatory T cell (Treg) formation and activity while reducing Th17 differentiation in vitro. This effect was mediated by the Notch signaling pathway through the expression of suppressive factors such as PGE2 [8–10]. PGE2 secretion was observed also to be induced following TLR-4 ligation through activation of the NF-κB signaling pathway [11].

The differential effects of TLR ligands on MSCs are associated with the downstream activation of multiple immunomodulatory signals. Similarly, IFN-γ, TNF-α, and IL-1β are released by activated immune cells in the inflammatory environment and can activate intracellular signaling pathways in MSCs, which results in the production of several molecules responsible for acquired immunomodulatory functions [12–14]. Accordingly, ex vivo licensing of MSCs improves MSC-mediated immune modulation in several animal models of inflammatory and autoimmune diseases through T-cell inhibition, M2 macrophage polarization, and Treg expansion [15–18]. Therefore, ex vivo/in vitro priming of MSCs using proinflammatory stimuli can be used to study the mechanism of MSC-derived immunomodulation and adopted as a strategy to enhance the therapeutic target profile of MSCs.

Mechanistically, IFN-γ- and TNF-α-induced licensing induces MSC expression of immunomodulatory factors through the JAK-STAT1 signaling pathway, which results in the suppression of T-cell proliferation [19, 20]. On the other hand, IL-1β-primed MSCs exert immunosuppressive effects through the release of factors such as IL-6, TNF-α-stimulated gene 6 (TSG-6), and cyclooxygenase-2 (COX-2) via the NF-κB pathway [21, 22]. As a consequence, the combination of proinflammatory cues to which MSCs are exposed in the microenvironment and the resulting intracellular signaling cascade determine the range of molecules responsible for orchestrating the immunomodulatory activity [23]. The list of factors secreted by MSCs in response to the cytokine-dependent licensing is long and includes different cytokines, growth factors, and metabolic enzymes.

Among immunosuppressive cytokines, TGF-β is a potent factor released by MSCs with a broad activity that modulates immune cell functions. TGF-β primarily acts on antigen-presenting cells such as monocytes, macrophages, and dendritic cells (DCs) but also plays a role in T and NK cells. In vitro, TGF-β is secreted by MSCs, induces macrophage polarization toward the M2 phenotype, and improves the phagocytic ability of macrophages via activation of the Akt/FoxO1 pathway [24]. This phenomenon has been further explored in a mouse model of sepsis whereby the infusion of MSCs engineered to constituently overexpress TGF-β1 attenuated tissue damage, reduced the inflammatory response by decreasing macrophage infiltration in organs, and induced a phenotypic shift in macrophages from proinflammatory CD86+ cells to the pro-resolution CD206+ M2 phenotype [25]. Further in vitro studies reported that MSCs can promote the generation of Tregs through the release of TGF-β directly or indirectly through the induction of M2 macrophages [26–30], and this mechanism is crucial to reducing Th2-driven allergic responses in a mouse model of asthma [31, 32]. In addition, MSC-derived TGF-β inhibits CD8+ T-cell proliferation and function [33] and suppresses NK cell proliferation, cytokine secretion, and cytotoxicity through the induction of a regulatory senescent-like NK cell phenotype [34, 35]. Furthermore, MSCs suppress the expression of proinflammatory cytokines such as TNF-α by mast cells and inhibit B-cell maturation and IgE secretion in an atopic dermatitis mouse model, thus suggesting the potential of MSC treatment for allergic disorders [36].

IL-10 has also been described as one of the main cytokines involved in the immunosuppressive signaling of MSCs. Although its levels are low, IL-10 is expressed by other cell types in response to MSC-derived molecules such as PGE2 and IDO. For example, M2 macrophages that are polarized by MSCs secrete high levels of IL-10 [11, 37, 38]. In these conditions, autocrine IL-10 secretion by M2 macrophages further promotes the polarization of macrophages into an M2 phenotype, creating a positive-feedback loop [39]. Furthermore, MSCs induce a regulatory phenotype in DCs, that enables them to suppress T-cell activation and proliferation and promote Treg proliferation through IL-10 secretion [40, 41]. Given the potent immunomodulatory capacity, IL-10 is an appealing therapeutic target, and its overexpression in MSCs has proven of  therapeutic benefit as neuroprotective and anti-inflammatory in a rat model of ischemic stroke [42].

IL-6 represents a further cytokine in MSC immunosuppressive repertoire. MSC-derived IL-6 has been reported to delay apoptosis in lymphocytes and neutrophils [43, 44]. IL-6 mediates PGE2 release from MSCs and orchestrates subsequent immunosuppression in a preclinical model of arthritis. Accordingly, IL-6-deficient MSCs could not deliver therapeutic effects in this model [39]. Furthermore, the ability to inhibit DC differentiation and induce functional differentiation of monocytes toward IL-10-producing cells has been attributed to this cytokine [26, 39].

In addition to IL-6, HGF expressed by MSCs has been shown to inhibit monocyte differentiation into DCs and enhance the differentiation of mature DCs into regulatory DCs [39, 45]. Regulatory DCs have strong phagocytic activities and inhibit T-cell proliferation in vitro and in an acute lung injury model. MSCs engineered to overexpress HGF reduced DC accumulation and maturation in vivo [45]. HGF secreted from MSCs induces monocytes to produce high levels of IL-10, and this monocyte population suppresses activated CD4+ T-cell proliferation and modulates the T-cell cytokine profile from Th1 to Th2 [38, 39]. In addition, MSCs have been shown to modulate in vitro the conversion of fully differentiated Th17 cells into functional Tregs through an HGF-dependent mechanism, altering the Th17/Treg cell balance and reducing the levels of IL-17 and IL-6 but increasing the expression of IL-10 [46].

Importantly, the immunosuppressive MSC secretome includes IDO, which promotes the conversion of tryptophan into kynurenine, depleting the environment of an essential amino acid and favoring the accumulation of its toxic kynurenine metabolites. As a result, MSC-derived IDO suppresses immune cell activation and proliferation, which affects a wide range of targets, including T cells, B cells, NK cells, and DCs [12, 47–50] In addition, IDO activity promotes the differentiation of monocytes into M2 macrophages, resulting in IL-10 release [47]. In vivo, IDO increases the formation of Tregs and promotes immunological tolerance through a Th2 cytokine profile in a kidney allograft model [51, 52]. The IDO pathway is also prominently induced when MSCs undergo apoptosis in vivo and in vitro, which is a mechanism that is critical for their therapeutic function [53, 54].

PGE2 also plays an important role as an MSC effector molecule. PGE2 is constitutively secreted by MSCs at low levels, but its secretion increases in response to inflammatory mediators such as IFN-γ, TNF-α, and IL-1β [55, 56]. PGE2 induces M2 macrophage polarization in vitro and its effects have been demonstrated in a mouse model of sepsis, whereby is responsible for increasing IL-10 and decreasing TNF-α and IL-6 expression in macrophages [11, 57]. Similarly, coculturing MSCs with macrophages and mature DCs results in the polarization of macrophages and mature DCs toward an anti-inflammatory and phagocytic phenotype through the PGE2 axis, and the induction of this regulatory phenotype is abolished when PGE2 is inhibited [58]. PGE2 also exerts inhibitory effects on CD8+ T-cell proliferation and this has been confirmed using MSCs from multiple sources [59]. Furthermore, PGE2 suppresses Th17 cells in MSC-peripheral blood mononuclear cell (PBMC) cocultures, thus modulating the Th17/Treg balance [27]. This PGE2-dependent inhibition of CD4+ T cells differentiation into Th17 cells is consistent with previous studies showing that MSCs inhibit the production of IL-17, IL-22, IFN-γ, and TNF-α by fully differentiated Th17 cells via a mechanism that is at least partly mediated by PGE2 [60]. Finally, the inhibitory effects of MSCs on the proliferation and cytotoxicity of NK cells are dependent on PGE2 and IDO. Interestingly, there is evidence that this inhibition is increased when cells are in direct or close contact, suggesting the presence of additional mechanisms [50, 61].

Another soluble molecule with potent anti-inflammatory activities that is used by MSCs is TSG-6. TSG-6 is a 30 kD glycoprotein with anti-inflammatory properties that inhibits the TLR-2/NF-κB signaling pathway. It has been shown that the expression of this molecule correlates with the efficacy of MSCs in controlling sterile inflammation in mouse models of corneal injury, bleomycin-induced lung damage, and zymosan-induced peritonitis [62]. Interestingly, MSCs can self-activate caspase-dependent IL-1 signaling to enhance the secretion of TSG-6 [63], thus linking a therapeutically critical immunomodulatory molecule to the apoptotic cascade.

IL-1RA, a competitive inhibitor of IL-1α and IL-1β, has also been reported to be expressed in MSCs, and this factor inhibits the secretion of TNF-α by activated macrophages and promotes M2 polarization in vitro and in vivo. MSC-derived IL-1RA also suppresses CD4+ T‐cell activation and B‐cell differentiation [64–66].

MSCs have been shown to directly interfere with the function of cytotoxic cells. MSCs can target NK cell-mediated cytolysis and IFN-γ secretion by releasing soluble forms of HLA-G in an IL-10-dependent manner [67]. HLA-G, a non-classical HLA Ib molecule, is a specific ligand of KIR2DL4 (CD158d), which is mainly expressed by CD56^bright^/CD16^dim/−^ NK cells. Other HLA-G receptors include an immunoglobulin-like transcript (ILT)2 (CD85j, LILRB1), which is expressed by lymphoid cells. HLA-G also binds to CD160 on NK cells, endothelial cells, and T lymphocytes. As a result of these interactions, soluble HLA-G molecules inhibit the cytolytic functions of NK cells and T lymphocytes by downregulating perforin and Stat3 [68]. Finally, soluble HLA-G molecules interfere with the secretion of several chemokines by NK cells [69] and promote Treg expansion [67, 70].

Further mechanisms by which MSCs impair T-lymphocyte function involve direct cell–cell contact via receptor‒ligand interactions, such as through PD-1/PD-L1 signaling. This mechanism also induces M2-like polarization of monocytes and induces the expression of PD-L1 in macrophages [71–73]. In addition, by activating the Fas-FasL pathway, MSCs induce T-cell apoptosis, as demonstrated in an autoimmune model using FasL-knockout MSCs. The mechanism has been further elucidated in vitro through the transient knockdown of FasL, which revealed that activation of the Fas pathway induced MSC expression of MCP-1, promoting T lymphocyte recruitment and TGF-β expression in macrophages [74]. The transient effect of Fas-FasL pathway activation on T cells in a graft-versus-host disease (GvHD) model was improved by overexpressing FasL on MSCs in vitro and in vivo, suggesting an interesting therapeutic strategy for the treatment of GvHD [75, 76].

In addition to the classical cytokine-based licensing of MSCs, cell–cell contact mechanisms contribute to MSC activation and subsequent immunomodulation. Recent evidence demonstrated that activated cytotoxic cells mediate indirect immunomodulatory effects on MSCs by inducing them to undergo apoptosis [53]. As a result, apoptotic MSCs are phagocytosed by monocytes/macrophages, which acquire an anti-inflammatory phenotype [54]. This cytokine-independent pathway for the induction of M2 macrophage polarization has also been confirmed in vivo [77, 78]. This mechanism resolves the long-standing conundrum of why the majority of systemically infused MSCs rapidly disappear despite their long-lasting immunomodulatory and therapeutic effects. In fact, after initially being trapped in the lungs, most cells become undetectable within 24 h following their infusion [77, 79]. Importantly, the critical role of apoptosis is consistent with the several immunosuppressive molecules that have been identified in previous studies, and a large proportion of these factors can be directly or indirectly triggered after caspase activation. Finally, these findings further highlight the key role of monocytes/macrophages as the ultimate mediators of the immunosuppressive effects of MSCs [53].

## Regulated cell death and inflammation

The emerging concept that MSC-mediated immunomodulation is derived from the activation of cell death pathways informs novel molecular players and instructs new approaches to enhance MSC therapeutic efficacy. Importantly, it provides insights into the role of cell death-mediated immunomodulation in the context of MSC therapy and opens questions on the impact of different types of MSC death on the immune system.

It has long been shown that dying and dead cells are potent immunomodulatory agents in the microenvironment through a variety of mechanisms ranging from the intricate process of efferocytosis to the active secretion of immunomodulatory molecules. Their effects on the immune system are numerous and reflect the different ways in which cells initiate and execute their own death.

In general, cell death can occur in two ways: as a passive and unregulated process caused by tissue injury referred to as accidental cell death or as a result of highly regulated mechanisms involving different signaling cascades to elicit a variety of effector functions, which is referred to as regulated cell death (RCD). The accidental form of cell death, also known as necrosis, occurs when the cellular stress is so severe (e.g., highly toxic compounds, starvation, DNA damage) that the cell fails to maintain intracellular homeostasis. These conditions rapidly lead to cell swelling, rupture of the plasma membrane and the subsequent passive release of intracellular contents into the microenvironment, thus inducing inflammation and resulting in tissue damage.

In the case of RCD, the scenario is more complex. Many types of RCD have been described thus far, and their molecular signatures are well defined [80]. Generally, RCD can be classified as immunogenic or immune-silent based on its ability to trigger adaptive immune responses and contribute to the exacerbation or resolution of inflammation.

### Necroptosis and pyroptosis: the immunologically active forms of cell death

Necroptosis is a programmed form of necrosis initiated by the activation of death receptors such as Fas and tumor necrosis factor receptors (TNFR1 and TNFR2), Toll-like receptors (TLR-3 and TLR-4), and nucleic acid sensors in a context in which caspase activation is inhibited [81]. The necroptotic signaling cascade ultimately results in the activation of mixed lineage kinase domain-like pseudokinase (MLKL) and its insertion into the plasma membrane, which mediates membrane rupture and the leakage of intracellular contents [82].

Similar to necroptosis, pyroptosis also results in the loss of plasma membrane integrity [83]. Pyroptotic cell death is initiated by danger- or pathogen-associated molecular patterns (DAMPs or PAMPs), which interact with intracellular sensors to activate protein complexes called inflammasomes. Inflammasomes are responsible for activating caspase-1, which in turn cleaves and activates the proforms of the inflammatory cytokines IL-1β and IL-18. Caspase-1 further cleaves the gasdermin D (GSDMD) protein, which forms pores in the cell membrane, allowing the release of IL-1β and IL-18 [83].

Through the release of danger signals and cytokines from necroptotic and pyroptotic cells, the immune system is alerted to the potential danger and activates a large variety of innate proinflammatory pathways that result in the generation of innate and adaptive immunity, culminating in the establishment of immunological memory.

Interestingly, a recent study investigating the secretome of human myeloid cells undergoing TNF-induced apoptosis and necroptosis revealed that necroptotic but not apoptotic cells use lysosomal exocytosis to release intracellular contents [84]. This strategy, which had already been described as a membrane repair mechanism, could be a modality to communicate with immune cells and deliver proinflammatory cues before cell disintegration. In a different study, cytokine production continued in necroptotic cells following the loss of plasma membrane integrity, providing further evidence that dying cells contribute to the amplification of the inflammatory response through differently regulated mechanisms [85].

Activation of the immune system is a downstream effect of these types of RCD and is fundamental during microbial infections. In these circumstances, necroptosis and pyroptosis serve as mechanisms to enhance host defense [86–88]. Furthermore, immunogenic RCD has been demonstrated to be involved in the pathogenesis of a number of inflammatory diseases, as well as in the tumor microenvironment [89].

In the context of cancer, necroptotic cell death is strongly connected with antitumor immunity, and necroptotic cells have been explored as a treatment to fight cancer progression. Necroptotic cancer cells can potently activate DCs through the release of IL-1α and DAMPs in vitro [90, 91] and promote T-cell activation through robust cross-priming, resulting in tumor attack in vivo [92, 93]. In addition, the use of necroptotic cells as a vaccination strategy efficiently induced antitumor immune responses [91]. These studies demonstrated that the antitumor immune effect of necroptotic cells was derived mainly from the activation of the proinflammatory NF-κB pathway, indicating the substantial role of dying cell-derived proinflammatory molecules in stimulating immunity. Similarly, the induction of pyroptosis in tumors enhances the phagocytosis of pyroptotic cancer cells by tumor-associated macrophages and activates cytotoxic immune cells, thereby intensifying tumor immune responses [94]. Consistently, by using a biorthogonal chemical system, a different study demonstrated that the selective induction of pyroptosis in less than 15% of tumor cells in vivo resulted in massive tumor regression that was dependent on the infiltration of NK and T cells [95]. However, pyroptotic cell death can have the opposite effect on the tumor microenvironment. It has been suggested that pyroptosis in immune cells but not cancer cells is associated with tumorigenesis and cancer progression through the development of chronic inflammation and the inhibition of antitumor cytotoxicity [83].

To our knowledge, no studies have yet directly investigated the effect of necroptotic and pyroptotic MSCs on the immune system. Interestingly, MSCs appear to be resistant to pyroptosis following stimulation with conventional PAMPs and require a more complex environment enriched in soluble factors from pyroptotic macrophages [96]. On the other hand, early studies showed that MSCs activate the complement cascade and are rapidly eliminated after exposure to serum, thus raising concerns about whether this limited lifespan could result in impaired activity in vivo [97, 98]. It is likely that complement-mediated killing affects MSC-mediated immunosuppression by inducing inflammatory RCD [99]. In this regard, assessing the hemocompatibility of MSC products before intravascular infusion could be a valuable step forward in improving clinical efficacy [100].

### Apoptosis: the immune-silent form of cell death

Apoptosis is the most characterized type of RCD and has been investigated for over 30 years. Apoptotic cell death is initiated by triggers ranging from developmental cues to cellular stressors or cytotoxic immune cells and can be mediated by two distinct pathways: the intrinsic and extrinsic pathways [101]. The intrinsic pathway is activated when toxic substances compromise the intracellular environment, leading to mitochondrial damage. In these circumstances, proapoptotic proteins in the B-cell lymphoma-2 (BCL-2) family, such as BCL-2-associated X (BAX), BCL-2 homologous antagonist killer (BAK), and BCL-2-related ovarian killer (BOK), become activated and translocate to the mitochondrial membrane, forming pores and causing mitochondrial outer membrane permeabilization (MOMP) [102]. This event results in the rapid release of danger signals into the cytoplasm, such as cytochrome c and mitochondria-derived activator of caspases (SMAC), and the formation of a multiprotein complex called the apoptosome [103]. The apoptosome subsequently triggers the activation of the initiator caspase-9, which in turn cleaves several other procaspases and induces the caspase cascade [104, 105]. Furthermore, antiapoptotic proteins in the BCL-2 and BAX family, such as BCL-2 and BCL-XL and proteins belonging to the inhibitor of apoptosis (IAP) family (IAP1/2 and XIAP), act as intracellular inhibitors of apoptosis by sequestering the BAX and BAK complexes or interfering with the activation of caspases, respectively [106–108]. However, the release of SMAC from the mitochondria efficiently inhibits the activity of IAP proteins [109]. Thus, the fine, intricate balance among apoptotic proteins within the intracellular environment determines cell fate during intrinsic apoptosis.

In the extrinsic pathway, the initial apoptotic triggers are provided by the microenvironment and surrounding cells. Similar to necroptosis, extrinsic apoptosis is initiated by the binding of the cell death receptors TNFR1 and TNFR2, Fas, and the TNF-related apoptosis-inducing ligand (TRAIL) receptors DR4 and DR5 to their respective ligands TNF-α, FasL and TRAIL [101]. Upon binding, the receptors oligomerize and recruit additional molecules to form the death-inducing signalling  (DISC) complex. Depending on the initial trigger, the DISC complex exhibits distinct functions. In the case of stimulation by TNF-α, the DISC complex, which is also known as complex I, initiates survival activities through the NF-κB signaling pathway. However, when NF-κB and MAPK signaling are inhibited, a second complex is formed that activates the initiator caspase-8 and caspase-10, thus triggering the effector caspase cascade [110]. In some cell types, activation of the extrinsic pathway is not sufficient to trigger cell death, and the combination of intrinsic and extrinsic pathways is needed. In these cells, caspase-8 cleaves and activates the BH3 interacting-domain death agonist (BID), which activates BAX and BAK, promoting MOMP and the formation of the apoptosome [111].

Activation of the caspase cascade leads to an irreversible series of events that characterize the apoptotic process, which include cell shrinkage, membrane blebbing, DNA fragmentation, and the release of apoptotic bodies. Caspases are also responsible for the cleavage and activation of the enzyme Xkr8, which flips phosphatidylserine (PtdSer) to the outer leaflet of the plasma membrane, thus promoting the engulfment of apoptotic cells by professional phagocytes [112]. The entire apoptotic process maintains the integrity of the cell membrane and avoids the release of dangerous cellular components into the environment, thus causing minimal damage to the surrounding tissues.

Apoptosis was long considered an immune-silent mechanism of cell removal based on the fact that apoptotic cells do not trigger adaptive immune responses. It is now clear that apoptotic cells can actively engage with the immune system and elicit immunosuppression. A wide body of evidence identifies the efferocytosis of apoptotic cells by professional phagocytes as the main effector mechanism of apoptotic cell-driven immunomodulation. Indeed, the engulfment of apoptotic cells can educate phagocytes toward an anti-inflammatory phenotype [113–115], and this has been used as a therapeutic strategy to treat a number of inflammatory conditions [116].

In addition to the efferocytosis mechanism, apoptotic cells also directly contribute to immunosuppression by actively releasing anti-inflammatory and immunomodulatory molecules. For instance, apoptotic cells express or release several factors, including IL-10 [117], TGF-β [118], CCR5 [119], thrombospondin-1 [120], and Annexin1 [121], that can skew immune responses in favor of immunosuppression. A recent study characterized the metabolic profile of the apoptotic cell secretome and its effects on neighboring cells [122]. The authors showed that different apoptotic cell types release specific metabolites via a regulated mechanism that involves caspase-dependent pannexin 1 channels. The apoptotic secretome induced gene signatures related to inflammation, wound healing, and tissue repair in phagocytes and attenuated inflammation in models of arthritis and lung transplantation in vivo [122].

Mechanistically, at least some of the immunosuppressive effects of apoptotic cells are attributed to the activation of caspases. Several studies have demonstrated that these proteases can also have nonapoptotic effects, including anti-inflammatory effects. In the context of intrinsic apoptosis, the caspase cascade is required to inhibit mitochondrial DNA-induced type I interferon production [123, 124] and TNF-α secretion from dying cells [125]. Likewise, the activation of apoptotic caspases is responsible for the neutralization of high-mobility group box-1 protein (HMGB1) stimulatory activity [126]. These studies revealed that caspases in the intrinsic pathway are not essential for cell death to occur; they instead guarantee that the apoptotic process is not immunogenic by suppressing proinflammatory cytokine release from dying cells.

## The paradigm shift: MSC apoptosis as critical mechanism of immunosuppression

The long-standing hypothesis of the immunomodulatory effects of MSCs being dependent on viable cells is now challenged by the emerging evidence that cell viability is not critical and may be marginal to the therapeutic efficacy of MSCs. Early in vitro and in vivo studies indicated the effectiveness of apoptotic MSCs (ApoMSCs) in eliciting immunosuppression. Lu et al. initially observed that the supernatant from macrophages that had phagocytosed dead MSCs significantly improved the survival of cardiomyocytes in hypoxic conditions [127]. Moreover, in a rat sepsis model, the injection of ApoMSCs improved survival and reduced the levels of plasma TNF-α and circulating Th1 cells [128]. This principle has been further explored, and the efferocytosis of ApoMSCs has been shown to shift macrophages toward an M2 phenotype, thereby reducing TNF-α and nitric oxide (NO) production while increasing IL-10 secretion [129].

We have previously demonstrated that cytotoxicity, which is measured by the ability to induce apoptosis in MSCs, is not only critical for the immunosuppressive effects of MSCs but is also predictive of the therapeutic benefit of MSCs in patients affected by severe steroid-refractory GvHD [53]. Further mechanistic evidence was generated in a mouse model of GvHD and corroborated by recent in vitro studies and has demonstrated that, following efferocytosis of ApoMSCs, macrophages upregulate and secrete molecules such as PGE2, PD-L1, IL-10, and IDO [54]. In a comparable study, viable umbilical cord-derived MSCs were identified in the lung following systemic infusion and were phagocytosed by monocytes and redirected to the bloodstream and the liver. The phagocytosis of MSCs induced monocyte reprogramming to an M2 phenotype and induced Treg cell formation in a mixed lymphocyte reaction in vitro [77].

Similar effects of ApoMSCs have also been demonstrated in a mouse model of asthma in which injection of live and ApoMSCs decreased eosinophil infiltration and lung tissue inflammation [130]. In contrast to our previous data, live MSCs were also cleared from the lungs of recipient mice that were genetically deficient for T, B, and NK cells, suggesting that MSC apoptosis can occur in the absence of some cytotoxic cells in vivo. However, macrophages and granulocytes, which persisted in this model, can also secrete cytolytic granules and possibly replace the absence of conventional cytotoxic immune cells [131, 132]. The study corroborated the evidence that the rapid clearance of ApoMSCs from the lung is driven by phagocytic cells such as neutrophils and monocytes/macrophages [130]. Most recently, a new therapeutic strategy of inducible apoptosis in MSCs was tested in a preclinical animal model of inflammatory bowel disease. Inducible caspase-9 was introduced into MSCs as a suicide gene switch and was activated 8 h after MSC injection. Although survival was superior in the live MSC group, induced ApoMSCs generated similar levels of infiltrating leukocytes and serum levels of proinflammatory cytokines, indicating a similar mechanism of action and the use of inducible apoptosis as a potential MSC therapy [133].

In light of the critical role of MSC apoptosis in achieving therapeutic efficacy, it is interesting to observe that almost all the inflammatory molecules that contribute to MSC licensing have been shown to be strictly involved in the induction of apoptotic pathways. For instance, in addition to the well-known proapoptotic activity of TNF-α, IFN-γ can sensitize cells to apoptotic stimuli by upregulating many apoptosis-related genes, such as *FAS* and interferon-regulatory factor 1 (*IRF1*) [134, 135]. Moreover, TLR activation can induce apoptosis via different mechanisms, including myeloid differentiation factor 88 (MyD88)-mediated FADD and caspase-8 signaling [136], molecular adaptor Toll/IL-1R domain-containing adapter inducing IFN-beta (TRIF) signaling [137], and the dsRNA-dependent protein kinase (PKR)-induced death pathway [138]. Collectively, these data suggest a link between cytokine-dependent licensing and the induction of cell death, whereby apparently distinct mechanisms drive a common downstream effector function.

## Stromal cell apoptosis as a new therapeutic target profile

The promise of the beneficial multitasking activities of MSCs and the ease of their isolation for GMP manufacturing have attracted much attention and resulted in several studies that tested MSCs for the treatment of several types of disease. Unfortunately, clinical successes have been very limited. One of the main reasons is that clinical trials were executed before obtaining a thorough understanding of the mechanism of action, the disease target profile, and the criteria for patient selection. As of the time of the preparation of this manuscript, 1777 clinical trials have been recorded in the context of inflammatory diseases; 547 were completed, but only 40 were in phase 3. It is not surprising that such an inconclusive experience has been discouraging and somehow detracted from the real value of MSCs as therapeutic agents. There is now more information to reinterpret the available results and propose new approaches to focus on clinical conditions that may truly benefit from MSCs.

One of the major insights produced in the last few years is MSC “licensing”, which is the need for MSCs to be exposed to the appropriate inflammatory microenvironment to acquire their immunomodulatory and therapeutic properties. A better understanding of the correct cues to elicit such a response can provide an invaluable molecular classifier to identify disease target profiles and stratify patients for treatment. Although much information is available about the stimuli that trigger MSC immunosuppressive activity, these findings have rarely been applied to clinical practice. We have extensively discussed the roles of TLR ligands and cytokines that enable MSC properties. A combination of these licensing factors plays a critical role in the pathogenesis of different conditions and is likely to impact MSC functions and clinical responses. The concentration and type of these molecules also change during the course of the disease, highlighting the importance of profiling not only the disease but also the disease stage at which patients receive MSCs. A very interesting clinical study was performed on 105 patients affected by persistently active rheumatoid arthritis who had failed standard treatment. The patients were randomized to receive MSCs or placebo. Of the 52 patients who received MSCs, the 28 who were classified as responders exhibited a transient increase in serum IFN-γ (>2 pg/ml) levels in comparison with those who did not respond [139]. These data are consistent with the well-established link between IFN-γ and the upregulation of IDO in MSCs. Another study examined MSCs for treating GvHD in 10 patients and showed that mean plasma levels of interleukin 2 receptor alpha (IL-2Rα) and TNFR1 in acute GvHD patients before MSC infusion were high in responders and persistently decreased after MSC treatment [140].

The recent discovery that in vivo MSC apoptosis plays a critical role in the therapeutic activity in GvHD and that this process is mediated by activated cytotoxic cells has provided novel insights into patient selection for treatment. The detection of anti-MSC cytotoxic activity in patient peripheral blood is associated with clinical responses to MSCs [53] and may represent a unique and reliable biomarker to stratify patients for treatment [141]. We examined the in vitro cytotoxic activity of PBMCs obtained from 31 steroid-resistant acute GvHD patients collected the day before MSC treatment. We found that PBMCs from responders exhibited significantly higher cytotoxicity against MSCs than those from non-responders and that the cytotoxic assay was predictive of response with 91.7% sensitivity and 90.0% specificity. Increased cytotoxicity was significantly associated with clinical response and median overall survival in multivariate logistic regression analysis (*p* < 0.001) [142].

Therefore, a focus on selecting diseases characterized by the infiltration of cytotoxic T cells, NK cells, or other innate lymphoid cells could be a sensible starting point. This is a frequent occurrence not only in alloimmune conditions such as GvHD and graft rejection but also in autoimmune disorders such as inflammatory bowel disease [143], multiple sclerosis [144], and psoriasis [145]. Furthermore, several sterile, nonimmunological tissue injuries are characterized by the infiltration of cytotoxic cells. For example, a persistent cardiac T-cell response initiated by myocardial infarction is linked to subsequent adverse ventricular remodeling and the progression of heart failure [146]. Similarly, CD8+ T and NK cells infiltrate the brain with different dynamics following ischemic stroke [147].

The currently available data strongly suggest that, rather than concentrating on characterizing the properties of MSCs that might not be relevant to their therapeutic efficacy, such as their differentiation ability or non-specific surface markers, the path to deliver effective immunomodulatory therapeutic effects should be developed around our understanding of the complex molecular and cellular milieu of target diseases and how this milieu varies among patients and across disease stages. Such an approach will enable the best patient selection and enhance the impact on clinical outcomes.

## Stromal cell apoptosis as an innate mechanism of tissue repair

It is well established that apoptosis is a key mechanism in normal tissue homeostasis. Apoptosis controls cell numbers in rapidly regenerating organs [148], regulates development in which cell overproduction is required for fine tuning [149], and contributes to correct tissue healing [150]. In the context of tissue injury, the efferocytosis of apoptotic cells can effectively reprogram phagocytic cells toward a pro-resolution phenotype that is essential for driving tissue repair, which is the case in every tissue. For example, a specific subset of macrophages identified as CD11b^hi^F4/80^int^LY6C^low^ cells can regulate tissue remodeling following liver injury. These restorative macrophages have increased the production of matrix metalloproteinases (MMPs) and decreased expression of proinflammatory cytokines and chemokines, and their depletion in vivo impaired healing in a model of hepatic fibrosis. Importantly, the authors demonstrated that these macrophages derive from proinflammatory monocytes that undergo a phenotypic switch after phagocytosis [151]. In a recent study, Vagnozzi et al. demonstrated that intracardiac injection of dying cells enhanced heart function after ischemia‒reperfusion injury. The beneficial effect was achieved through the induction of a specific subset of macrophages in the heart, which was accompanied by a reduction in the extracellular matrix in the injured area and the activation of cardiac fibroblasts [152]. In a separate study, efferocytosis of apoptotic cells upregulated MYC via activation of the mTOR2/Rictor pathway and promoted the proliferation of TGF-β- and IL-10-secreting macrophages in mouse models of peritonitis and atherosclerosis regression. In vivo silencing of the Rictor pathway blocked the expansion of proresolving macrophages and impaired tissue repair after injury [153]. In addition to the extensive data on the functional reprogramming of macrophages after the efferocytosis of apoptotic cells, further evidence reveals a similar effect on other types of professional and nonprofessional phagocytes. For instance, enhanced efferocytosis of dying cells by phagocytic DCs accelerated the healing of chronic wounds in a mouse model of type 2 diabetes [154]. Likewise, dermal fibroblasts acquired a pro-healing phenotype after the engulfment of apoptotic cells [155].

A separate body of evidence further reveals the substantial contribution of apoptosis to the normal homeostasis of tissues. It has become clear that apoptotic cells not only educate phagocytes to become anti-inflammatory and restorative cells but also engage with other neighboring cells in the tissue through caspase-dependent signaling. Apoptotic cells release caspase-dependent mitogenic factors and trigger a process called apoptosis-induced proliferation (AiP) in surrounding cells, confirming the crucial impact of apoptosis on tissue remodeling after injury. Highlighting the importance of such a mechanism is the fact that AiP is conserved among different organisms. In Drosophila, AiP is mediated by different signaling cascades that are controlled by initiator and executioner caspases [156, 157]. In mice, the release of growth signals from apoptotic cells stimulates the proliferation of progenitor and stem cells and promotes wound healing and tissue regeneration. Mechanistically, regeneration is accomplished via the production of caspase-3- and caspase-7-dependent PGE2 by apoptotic cells [158]. A similar role of AiP has been described within the tumor microenvironment. Caspase-3-dependent PGE2 release by apoptotic cancer cells was shown to promote tumor repopulation after chemotherapy and radiotherapy [159, 160]. Of note, in vivo administration of COX-2 inhibitors was sufficient to decrease the recurrence of chemoresistance in treated animals [160]. Furthermore, new studies shed light on additional AiP-related molecular pathways. Ankawa et al. demonstrated that apoptotic hair follicle stem cells (HFSCs) contributed to skin healing and regeneration after injury by inducing WNT3 expression. The authors observed that caspase-9 deletion in HFSCs slowed the apoptotic process, resulting in the delayed clearance of apoptotic cells. Slowly dying HFSCs had increased activation of capsase-3 and produced high levels of Wnt3, which induced stem cell proliferation [161]. Consistent with these results, Wnt8a-containing apoptotic bodies released by dying stem cells were shown to enhance the proliferation and maintenance of normal homeostasis in epithelial tissues [162].

Taken together, these studies underscore the multiple roles of apoptosis in the remodeling of tissues after damage and reveal the importance of apoptotic cell death in the homeostasis of tissue repair.

**Fig. 2:**
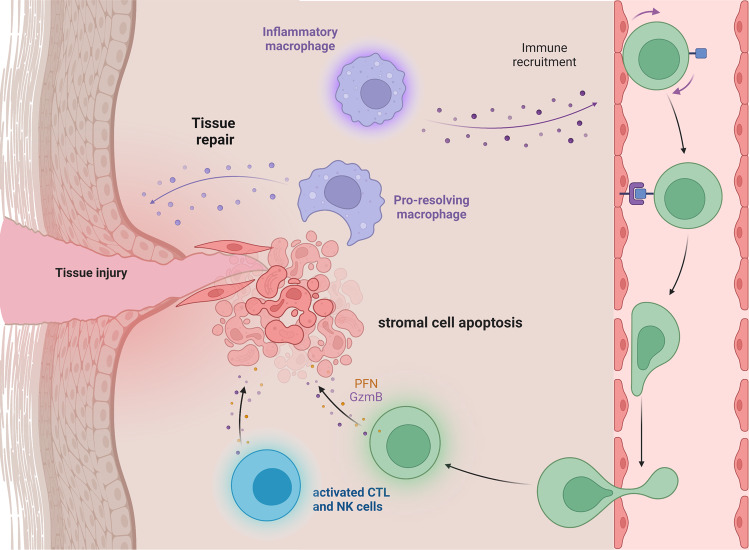
Stromal cell apoptosis as an innate mechanism of tissue homeostasis. Following injury, monocytes and macrophages rapidly migrate to the site of injury, where they become activated and start recruiting additional immune cells. Subsequently, the tissue is infiltrated by CTLs and NK cells that release perforin/granzyme B-loaded cytolytic granules to induce the surrounding tissue stromal cells to undergo apoptosis. The phagocytosis of apoptotic stromal cells efficiently reprograms macrophages into a pro-resolving phenotype that orchestrates the resolution of inflammation and tissue repair responses. Figure generated using BioRender.com

The emerging evidence that the therapeutic activity of MSCs is mediated through apoptosis raises the question of whether such a mechanism has physiological importance. Phenotypically and functionally, MSCs exhibit striking similarities to tissue fibroblasts [163], which are present in virtually every tissue of the body, although in different versions and subsets. Furthermore, following a sterile inflammatory lesion of any nature, the injured tissue is infiltrated by NK cells and cytotoxic T lymphocytes (CTLs) that are loaded with cytolytic granules. This infiltration is associated with the migration of monocytes/macrophages to the site of injury, suggesting that their reprogramming through the phagocytosis of cytotoxic cell-induced apoptotic stromal cells may represent a physiological event that controls inflammation and consequently prompts tissue repair (Fig. 2).

There are a few indirect pieces of evidence supporting this possibility. The failure of CTLs or NK cells to kill target cells by perforin/granzyme-induced apoptosis causes severe immune dysregulation. Early studies have shown that perforin deficiency is associated with macrophage activation syndrome and refractory systemic inflammation [164]. Furthermore, in familial hemophagocytic lymphohistiocytosis, perforin-deficient infants suffer a fatal cytokine storm due to macrophage overactivation. Similarly, it has been observed that in response to in vitro activation, perforin-deficient chimeric antigen receptor (CAR)-T cells produce higher amounts of proinflammatory cytokines than wild-type CAR T cells [165]. In a mouse model of nonalcoholic steatohepatitis, the disease was more severe in perforin-deficient mice than in wild-type mice fed a high-fat diet. Perforin deficiency was associated with the M1 polarization of infiltrating monocytes and an increase in proinflammatory cytokines in CD8+ T cells [166].

Although these data suggest that T cells directly control macrophage activation, the hypothesis that activated cytotoxic T or NK cells also provide apoptotic cells for functional reprogramming in macrophages is a plausible complementary mechanism. Accordingly, it has been shown that the adoptive transfer of fibroblast activation protein α (FAPα)-specific CAR T cells reduces cardiac fibrosis and abnormal cardiac remodeling and restores cardiac healing and function [167]. Although fibroblast depletion plays a critical role, it cannot be excluded that CAR-T-induced apoptotic fibroblasts also contribute to healing by reprogramming a deranged inflammatory environment.

The ultimate role of macrophages in restoring tissue homeostasis via ApoMSCs supports our hypothesis and has been confirmed in other studies. In the context of liver injury, recent evidence has demonstrated that MSCs can alleviate liver fibrosis in vivo by inducing a phenotypic switch in macrophagess from profibrotic to proresolving via the release of IL-10 and IL-4. MSCs undergo extensive apoptosis after infusion and release large amounts of apoptotic bodies, which in turn are phagocytosed by restorative macrophages, resulting in MMP12 expression and further contributing to the resolution of liver fibrosis [168]. In a different study, Ko et al. reported that MSC-derived extracellular vesicles enhanced the phagocytic activity of macrophages and induced the expression of amphiregulin. MSC-educated macrophages could preserve tissue-specific stem cells, limit inflammatory immune responses by inducing Tregs and control tissue homeostasis in models of autoimmune and sterile injuries in vivo [169].

Finally, we have generated evidence that MSC apoptosis stimulated by activated cytotoxic cells is independent of the immunological synapse and is mediated by the release of cytotoxic granules, and MSCs behave as innocent bystanders [53]. Interestingly, we have observed that MSCs, as well as fibroblasts, are uniquely sensitive to these effects, while cells from other lineages are largely unaffected (Giacomini et al., manuscript in preparation). These data support the hypothesis that at least a subset of tissue stromal cells may be specifically designed to sense injury-associated inflammation. After undergoing apoptosis, stromal cells deliver signals to reprogram the negative inflammatory microenvironment, thereby orchestrating tissue repair and homeostasis.

## Concluding remarks

The encouraging safety profile of MSCs has not been matched by clear and consistent clinical efficacy. This discrepancy can be largely attributed to the poor insights into the mechanisms responsible for their therapeutic activity and the inability to determine reliable pharmacodynamics. Furthermore, the different culture conditions and preconditioning of MSCs to evoke their therapeutic properties have not been supported by any companion diagnostic assessment to provide information for release criteria and potency. Here, we have reviewed and proposed the concept that in vivo MSC apoptosis is a key driver of MSC-mediated immunosuppression and the restoration of homeostasis in injured tissues. This MSC licensing can be achieved by stimulation with inflammatory soluble molecules, as well as direct interactions with cytotoxic cells and results in potent activation of MSC immunomodulatory abilities through the production of an immunosuppressive secretome and the polarization of macrophages into anti-inflammatory/pro-regenerative cells following MSC efferocytosis.

Importantly, the MSC apoptosis cascade-based mechanism of action lends itself to stratifying patients for treatment, monitoring therapeutic efficacy, and informing new approaches to generate consistently effective MSCs. Overall, these new insights into the mechanism of MSC-derived therapeutic activity will prompt further preclinical investigations to support better designs of new MSC-based clinical trials. Ultimately, we hope that a greater understanding of the way ApoMSCs regulate immunomodulation and tissue repair will lead to the development of novel, safe, and efficacious MSC ATMPs for the treatment of these unmet clinical conditions.
